# Subepithelial Hyalinisation Predicts Recurrence of Unicystic Ameloblastomas

**DOI:** 10.3390/diagnostics12030756

**Published:** 2022-03-20

**Authors:** Dominic Augustine, Roopa S. Rao, Lakshminarayana Surendra, Bharti Gupta, Thuckanaickenpalayam Ragunathan Yoithapprabhunath, Pradeep Kumar Yadalam, Shazia Mushtaq, Zeeshan Hera Ahmed, Shankargouda Patil

**Affiliations:** 1 Department of Oral Pathology & Microbiology, Faculty of Dental Sciences, Ramaiah University of Applied Sciences, MSR Nagar, Bengaluru 560054, India; dominic2germain@gmail.com (D.A.); drroopasrao1971@gmail.com (R.S.R.); drsuri29@gmail.com (L.S.); 2 Department of Maxillofacial Surgery and Diagnostic Sciences, Division of Oral Pathology, College of Dentistry, Jazan University, Shwajra Campus, Jazan 45142, Saudi Arabia; drbhartigupta09@gmail.com; 3 Department of Oral and Maxillofacial Pathology, Vivekanandha Dental College for Women, Namakkal 637205, India; yoitha.dentist@gmail.com; 4 Department of Periodontics, Saveetha Institute of Medical and Technical Sciences, Saveetha Dental College and Hospitals, Saveetha University, Chennai 600077, India; pradeepkumar.sdc@saveetha.com; 5 Dental Health Department, College of Applied Medical Sciences, King Saud University, Riyadh 11451, Saudi Arabia; smushtaqdr@gmail.com; 6 Dental College Hospital, Medical City King Saud University, Riyadh 11472, Saudi Arabia; aheera@ksu.edu.sa

**Keywords:** ameloblastoma, unicystic, luminal, intra-luminal, mural, hyalinisation, recurrence

## Abstract

The inductive effect of hyalinisation and its influence on the biologic behaviour of ameloblastoma variants represent a scarcely researched domain of oral pathology. The complexity of the induction effects within the odontogenic apparatus, with the involvement of both ectodermal and mesodermal tissues, is responsible for diverse histopathological characteristics, hyalinisation being the major feature. The present study aims to deduce for the first time the correlation between the severity of hyalinisation (SOH) and recurrence in three unicystic ameloblastoma (UA) variants, namely, intra-luminal (UA-IL), luminal (UA-L) and mural (UA-M). Retrospectively diagnosed archival cases of UA-IL (*n* = 08), UA-L (*n* = 22) and UA-M (*n* = 30) were assessed for SOH and its correlation with recurrence. A subgroup comparison (between UA-IL/UA-L and UA-M) was also performed. The clinical parameters of the patients were also analysed from files for clinicopathological correlation with recurrence. Results: sub-epithelial hyalinisation (SEH) significantly correlated with the recurrence of UA-L and UA-M (*p* = 0.001). When the histologic types (UA-L and UA-IL vs. UA-M) were grouped and the correlation of SOH with recurrence was checked, it was observed that both groups (*p* = 0.001) showed strong statistical correlation. UA-M lesions with multilocular radiolucency (*p* = 0.001) also showed significant correlation with recurrence. SOH can be a reliable histological predictor of recurrence and of aggressive biologic behaviour in UA. The present study shows a significant association of hyalinisation with the biologic behaviour of UA. Further studies with immunohistochemical investigations could validate the presence of hyalinisation and identify the origin of the hyalinised product in UAs.

## 1. Introduction

Hyalinisation is a condition in which normal tissue deteriorates into a homogeneous, translucent material. The phenomenon of hyalinisation can be physiological or pathological [1]. Hyalinised tissue appears as pale or glassy, acellular and eosinophilic under conventional haematoxylin and eosin (H&E) staining [2]. The role of hyalinisation in odontogenic tumours has been a topic of considerable debate. 

Odontogenic tumours (OTs) are a diverse group of lesions varying from innocuous benign lesions, including hamartomas, to aggressive malignant tumours [3]. The varied clinical behaviour of lesions and their diverse histopathology hinders predicting prognoses. Solid multicystic ameloblastomas are more aggressive; the follicular pattern type has the highest recurrence rate of 29.5%, and the acanthomatous type has the lowest recurrence rate of 4.5% [4]. UAs are pathologic lesions characterised by a cystic morphology comprising an ameloblastomatous lining that may present as growth into the lumen with a fibrous capsule. Characterised by the proliferation pattern of the epithelial lining, they are classified into three histologic types, namely, UA-IL, UA-L and UA-M [5]. OTs reciprocatively induce changes between their epithelial competent and the ectomesenchymal stroma. Hyalinisation is a major product seen in tissues undergoing inductive changes; this further implicates the role of hyaline in OTs. Based on a literature review, it is evident that the role of hyalinisation in predicting the biologic behaviour of UA has not been evaluated. 

The current study tests the hypothesis that the presence of SEH favours recurrence in UA. The UA-L and UA-IL variants are assumed to be less aggressive with non-recurrent potential. [6] However, the correlation between SEH and recurrence in these lesions could change our perspective on the biologic behaviour of UA-L and UA-IL. UAs are usually treated with conservative management, often with “cyst” enucleation. There is literature evidence to indicate that UA-M variants are known to behave as conventional ameloblastomas and should be treated more aggressively [7].

The present study aims to assess the possible correlation between SEH and recurrence in three histologic variants of UA with clinicopathological correlation. The clinicopathological correlations obtained in this study could help predict the biologic behaviour of UA variants. This will ensure better treatment outcomes for patients by enabling the surgeon to rationally choose the best management option available.

## 2. Materials and Methods

### 2.1. Study Design

Ethical clearance was procured from the University Ethics Committee for Human and Animal Trials at MS Ramaiah University of Applied Sciences (MSRUAS), bearing number EC-2021/F/052. The study was performed on FFPE-diagnosed archival cases of UA, UA-IL (*n* = 08), UA-L (*n* = 22) and UA-M (*n* = 30) from the Department of Oral Pathology and Microbiology, Faculty of Dental Sciences. Clinical data comprising the biological parameters and patient details were retrieved and documented from the outpatient department. The retrieved tissues were heated to 55 °C for 10 min to facilitate deparaffinisation, followed by a xylene dip. The sections were stained with H&E stain for analysis. 

### 2.2. Histopathological Correlation of Hyalinisation in UA with Recurrence

The slides were coded randomly and examined by two clinical pathologists (R.S.R. and D.A.) to evaluate the SOH; in ambiguous cases, a third pathologist (S.L.) assessed the histopathological features of hyalinisation and its severity. The histologic type of UA was also noted and tabulated. The correlation between SOH and recurrence in UA was statistically analysed. A subgroup comparison (between intraluminal and luminal–mural type) was also performed.

### 2.3. Clinicopathological Correlation of UA with Recurrence

The clinical parameters of size (1–2 cm = 1, 3–4 cm = 2, >4 cm = 3), jaw involved (maxilla/mandible), site, radiographic features (multilocular/unilocular), cortical expansion and root resorption were analysed for statistical correlation with recurrence. 

### 2.4. Interpretation and Analysis

The SOH was noted in the cases of UA and scored (0 = absent, 1 = mild, 2 = moderate and 3 = intense). Photomicrographs of the slides were captured at ×100 magnification using a research microscope. The histopathological and clinicopathological parameter scores were analysed for statistical significance by employing the chi-square test (SSPS software, IBM, Amonk, NY, USA).

## 3. Results

### 3.1. Correlation of Histological Parameter with SOH

The descriptive features of UAs (*n* = 60) are shown in Table 1. 

**Figure 1:**
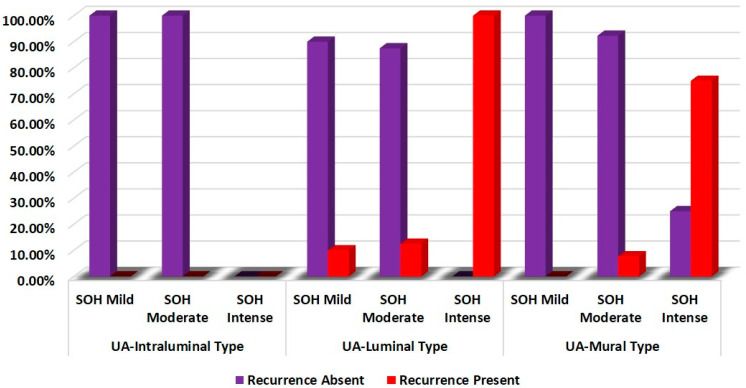
Comparison of correlation of SOH with recurrence of unicystic ameloblastoma.

SOH showed a significant statistical correlation with the recurrence of UA-L and UA-M (*p* = 0.001) (Table 2 and Figure 1).

**Figure 2:**
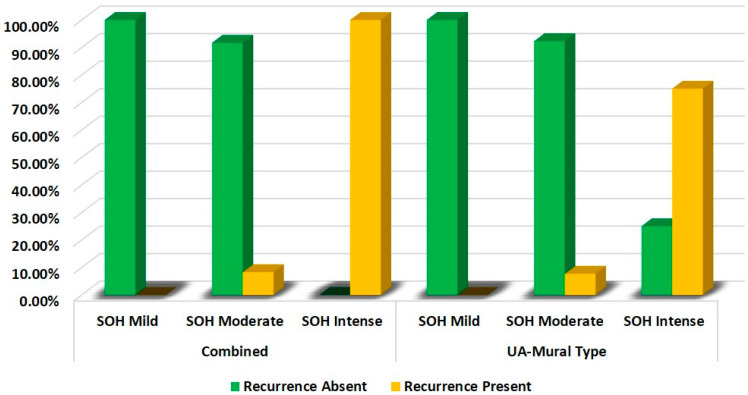
Comparison of correlation of SOH with recurrence of unicystic ameloblastoma combined (intraluminal/luminal) vs. mural type.

It was observed that both variants (UA-IL/UA-L vs. UA-M) showed strong statistical correlation between SOH and recurrence (*p* = 0.001), Table 3 and Figure 2. 

This indicates that the SOH influences the biologic behaviour of UA irrespective of the histologic subtype. 

### 3.2. Correlation between Clinical Parameters and Recurrence

It was noted that only UA-M lesions with multilocular radiolucency (*p* = 0.001) showed strong statistical correlation with recurrence (Table 4).

## 4. Discussion

Hyalinisation is the process of the deposition of hyaline, which is a homogenous, structureless, eosinophilic material reported to be present in oral benign and malignant lesions [7]. The term UA refers to lesions that are cystic and exhibit clinical/radiographic characteristics of a jaw cyst but on histopathological analysis show a typical ameloblastic epithelium satisfying “Vickers and Gorlin criteria” lining the cystic cavity, with or without luminal and/or mural tumour growth [8].

A series of progressive interplay is seen in the enamel organ between the epithelium and the connective tissue mesenchyme, which is derived from the neural crest during odontogenesis, resulting in inductive changes [9]. The role of hyalinised stroma in head and neck lesions has recently gained the attention of researchers owing to the developing concepts of significant epithelial–mesenchymal interactions. Considerable research is now ongoing to ascertain the involvement of hyalinisation in the biologic behaviour of odontogenic lesions. 

Desmoplastic reaction is paucicellular and is associated with benign and malignant tumours characterised by the pervasive growth of dense fibrous tissue around the tumour. The characterisation of desmoplastic reaction (D.R.) has emerged as a new, independent prognostic determinant in several cancers, such as colorectal and pancreatic cancers. Desmoplastic reaction is considered mature or immature based on whether the stroma is myxoid, and whether it contains or does not contain keloid such as collagen [10]. Hyalinisation is a process of conversion of stromal connective tissue into a homogeneous, acellular translucent material. It could be a degenerative change, a stromal response or even a secretion.

Cottom et al., in 2012, demonstrated a strong correlation between SEH and recurrent odontogenic keratocysts. The authors concluded that the presence of subepithelial hyalinisation may be used as an additional histopathological feature to predict a greater tendency for the recurrence of OKCs. This was the first report on the presence of hyalinisation and recurrence of the OKC [11]. Similar findings have been reported in salivary gland tumours of the head and neck; Katabi et al., in 2018, stated that prominent hyalinisation is a high-risk factor for the malignant transformation of pleomorphic adenomas [12]. The above findings provide adequate evidence to further explore the role of hyalinised stroma in other odontogenic tumours. 

**Figure 3:**
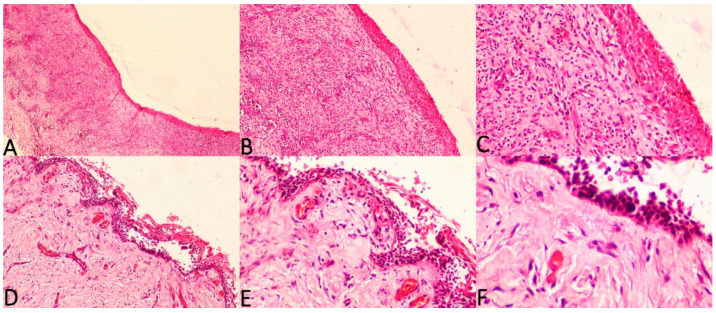
Photomicrographs of haematoxylin and eosin stain. Unicystic ameloblastoma—intraluminal type with absence of SEH, (**A**) 40×, (**B**) 100× and (**C**) 200×. Unicystic ameloblastoma—intraluminal type with prominent SEH, (**D**) 100×, (**E**) 200× and (**F**) 400×.

**Figure 4:**
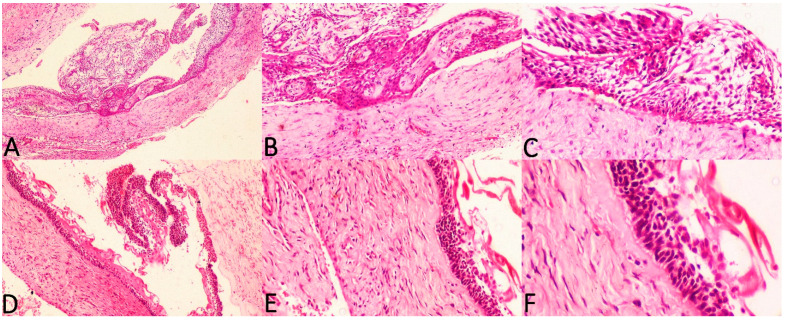
Photomicrographs of haematoxylin and eosin stain. Unicystic ameloblastoma—luminal type with absence of SEH, (**A**) 40×, (**B**) 100× and (**C**) 200×. Unicystic ameloblastoma—luminal type with prominent SEH, (**D**) 100×, (**E**) 200× and (**F**) 400×.

**Figure 5:**
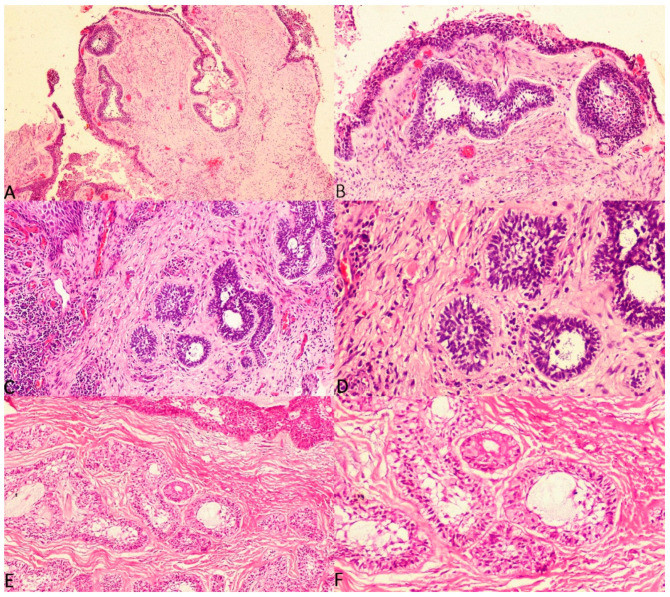
Photomicrographs of haematoxylin and eosin stain. Unicystic ameloblastoma—mural type with mild SOH, (**A**) 40× and (**B**) 100×. Unicystic ameloblastoma—mural type with moderate SOH, (**C**) 100× and (**D**) 200×. Unicystic ameloblastoma—mural type with intense SOH, (**E**) 100× and (**F**) 200×.

The current study considered three histologic subtypes of UA and attempted to draw an association between hyalinisation and recurrence aided by clinicopathological correlation. The SOH was compared between the subtypes of UA (UA-IL (Figure 3A–F), UA-L (Figure 4A–F) and UA-M); it was observed that a significant statistical correlation existed between SOH and recurrence in UA-M type (*p* = 0.001), Figure 5A–F. 

The presence of hyalinisation in odontogenic lesions represents the hyperactivity of the odontogenic epithelium, where the tumour cells are attempting to signal the connective tissue to form dental hard tissue. However, the odontogenic stroma fails to form hard tissue, instead resulting in an inductive change characterised by hyalinisation rich in proteins, glycosaminoglycans and hyaluronic acid [12,13,14,15,16]. Integrins play a pivotal role in transforming the extracellular matrix. They are adhesion molecules and mediate interactions between cells and the extracellular matrix. Integrins provide a mediation for cross-talks between tumours and their microenvironment, and facilitate a variety of changes such as hyalinisation [17]. It can be inferred that the hyalinised odontogenic stroma witnessed in solid multicystic ameloblastomas (SMAs) is indicative of the hyperactivity of the odontogenic cells, and such lesions can be expected to exhibit an aggressive biologic behaviour. The same hypothesis holds good for UA-M comprising odontogenic islands in the connective tissue stroma that behaves biologically similarly to SMA despite being a UA variant. 

In the UA-L and UA-IL variants, the presence of SEH is thought to be the secretory product of the lining cells, and the presence of hyalinisation does not seem to influence its biological behaviour [18]. It is postulated that the SEH zones adjacent to the basement membranes in UA contain large amounts of heparan sulphate. Sathi et al., in 2008, stated that heparan sulphate can prevent tumour growth and stop stromal–tumour cell interaction, resulting in the inhibition of the function of heparanase and angiogenesis. Finally, tumour cells adjacent to the hyalinisation undergo programmed cell death. However, the analysis was conducted on a sample of six cases of SMA [19]. 

Although the current study shows a statistical correlation (*p* = 0.001) between SOH and recurrence in UA-L type, we suggest that this be overlooked, since only 4 recurrent UA-L cases were available compared to 18 nonrecurrent UA-L cases in the present study. The smaller sample of recurrent UA-L is a limitation of the present study. Even though only four UA-L were recurrent, it is of considerable interest to note that the SOH was intense in all four recurrent cases of UA-L. 

UAs demonstrate a 6.7–35.7% recurrence rate in contrast to the 70–80% recurrence rate of SMAs following initial conservative therapy. In UA-M, the high recurrence rate is similar to that of solid or conventional ameloblastoma. Hence, in UA-M, the treatment is not limited to enucleation and curettage [20,21] It is believed that UA-IL and UA-L have a better prognosis than that of SMA, as the tumour cells are limited to the lining area and can be easily enucleated since the tumour cells do not invade the stroma. The same does not hold true in the case of UA-M, where ameloblastomatous islands invade the connective tissue, making UA-M behave more like a solid tumour and rendering its treatment challenging. 

A study by Cadavid et al., in 2019, evaluated the prevalence and clinical features of 136 ameloblastomas. The authors stated that the treatment of choice for SMA was segmental resection (45%), followed by curettage with cryotherapy (40%), with the remainder (15%) being treated with curettage alone. UA-IL and UA-L were treated conservatively by enucleation with curettage or cryotherapy [22,23]. SMA and UA-M required more invasive therapy. The authors concluded that owing to its higher rate of recurrence, UA-M is treated in the same manner as conventional SMA. 

When the clinicopathological features of size, jaw involved, site, radiographic features, cortical expansion and root resorption were correlated with UA-IL, UA-L and UA-M, no correlation was observed in the UA-IL and UA-L category. However, UA-M showed significant statistical correlation between the presence of multilocular radiolucency and recurrence. Lesions that are multilocular represent different growth sites of an infiltrative lesion. 

## 5. Conclusions

Despite the fact that UA compares favourably with SMA in terms of clinical behaviour and response to therapy, the subsets of multilocular lesions, UA-mural type and UA-L with intense SOH, could have a high risk of recurrence. The role of integrins and the mechanism of cross-talk induced to favour hyalinisation is a future area to investigate in odontogenic tumours. The presence of intense hyalinisation in subtypes of UA should be considered as an indicator of aggressive behaviour in UAs when determining the prognosis. This rational consideration by surgeons can help in deciding on the best therapeutic option to advocate to the patient.

## Figures and Tables

**Table 1:** Descriptive features of participants with unicystic ameloblastoma.

Characteristics	Frequency	Percentage (%)
**Age Groups**		
0–20	9	15.0
21–40	28	46.7
41–60	18	30.0
>61	5	8.3
**Gender**		
Male	38	63.3
Female	22	36.7
**Size**		
1–2 cm	14	23.3
3–4 cm	14	23.3
>4 cm	32	53.3
**Region**		
Body of Mandible	22	36.7
Angle of Mandible	29	48.3
Symphysis	8	13.3
Retromolar Trigone	0	0.0
Maxillary Sinus	1	1.7
**Jaw**		
Maxilla	1	1.7
Mandible	59	98.3

**Table 2:** Comparison of correlation of SOH with recurrence of unicystic ameloblastoma.

Groups	SOH	Recurrence		χ^2^	*p*-Value
		Absent	Present		
**UA-Intraluminal Type**	**Mild**	**100.0%**	0.0%	-	-
	**Moderate**	100.0%	0.0%		
	**Intense**	-	-		
**UA-Luminal Type**	**Mild**	90.0%	10.0%	15.950	**0.001**
	**Moderate**	87.5%	12.5%		
	**Intense**	0.0%	100.0%		
**UA-Mural Type**	**Mild**	100.0%	0.0%	14.024	**0.001**
	**Moderate**	92.3%	7.7%		
	**Intense**	25.0%	75.0%		

Chi—squared test, *p*-value < 0.05—statistically significant.

**Table 3:** Comparison of correlation of SOH with recurrence of UA—subgroups.

Groups	SOH	Recurrence		χ^2^	*p*-Value
		Absent	Present		
**Combined** **(UA-Intraluminal and** **UA-Luminal Type)**	**Mild**	100.0%	0.0%	22.067	**0.001**
	**Moderate**	91.7%	8.3%		
	**Intense**	0.0%	100.0%		
**UA-Mural Type**	**Mild**	100.0%	0.0%	14.024	**0.001**
	**Moderate**	92.3%	7.7%		
	**Intense**	25.0%	75.0%		

Chi—squared test, *p*-value < 0.05—statistically significant.

**Table 4:** Comparison of correlation of clinical parameter with recurrence of unicystic ameloblastoma—UA-mural type.

Clinical Parameters		Recurrence		χ^2^	*p*-Value
		Absent	Present		
**Age Groups**	0–20	33.3%	66.7%	4.072	0.254
	21–40	60.0%	40.0%		
	41–60	70.0%	30.0%		
	>61	0.0%	100.0%		
**Gender**	Male	55.6%	44.4%	0.023	0.880
	Female	58.3%	41.7%		
**Region**	Body of Mandible	50.0%	50.0%	3.529	0.171
	Angle of Mandible	50.0%	50.0%		
	Symphysis	100.0%	0.0%		
	Retromolar Trigone	-	-		
	Maxillary Sinus	-	-		
**Size**	1–2 cm	57.1%	42.9%	3.202	0.202
	3–4 cm	28.6%	71.4%		
	>4 cm	68.8%	31.2%		
**Jaw**	Maxilla	-	-	-	-
	Mandible	56.7%	43.3%		
**Radiographic** **Features**	Unilocular Radiolucency	85.0%	15.0%	19.615	**0.001**
	Multilocular Radiolucency	0.0%	100.0%		
**Cortical** **Expansion**	Absent	66.7%	33.3%	0.305	0.580
	Present	54.2%	45.8%		
**Root Resorption**	Absent	64.3%	35.7%	0.621	0.431
	Present	50.0%	50.0%		

Chi—squared test, *p*-value < 0.05—statistically significant.

## Data Availability

Not applicable.

## References

[1] von Böhl M., Kuijpers-Jagtman A.M. (2009). Hyalinization during orthodontic tooth movement: A systematic review on tissue reactions. Eur. J. Orthod..

[2] Chan J.K. (2014). The wonderful colors of the hematoxylin-eosin stain in diagnostic surgical pathology. Int. J. Surg. Pathol..

[3] Wright J.M., Soluk Tekkesin M. (2017). Odontogenic tumours: Where are we in 2017?. J. Istanb. Univ. Fac. Dent..

[4] Masthan K.M., Anitha N., Krupaa J., Manikkam S. (2015). Ameloblastoma. J. Pharm. Bioallied. Sci..

[5] Li T.J., Wu Y.T., Yu S.F., Yu G.Y. (2000). Unicystic ameloblastoma: A clinicopathologic study of 33 Chinese patients. Am. J. Surg. Pathol..

[6] Kim J., Nam E., Yoon S. (2017). Conservative management (marsupialization) of unicystic ameloblastoma: Literature review and a case report. Maxillofac. Plast. Reconstr. Surg..

[7] Saluja T., Iyer J. (2017). Unmasking the Grey Zone of Hyalinization with a Proposed Classification of Oral Hyalinizing Lesions. J. Interdiscip. Histopathol..

[8] Singh A., Shaikh S., Samadi F.M., Shrivastava S., Verma R. (2011). Maxillary unicystic ameloblastoma: A review of the literature. Natl. J. Maxillofac. Surg..

[9] Tummers M., Thesleff I. (2009). The importance of signal pathway modulation in all aspects of tooth development. J. Exp. Zool. Part B Mol. Dev. Evol..

[10] Ueno H., Ishiguro M., Nakatani E., Ishikawa T., Uetake H., Murotani K., Matsui S., Teramukai S., Sugai T., Ajioka Y. (2021). Prognostic value of desmoplastic reaction characterization in stage II colon cancer: Prospective validation in a Phase 3 study (SACURA Trial). Br. J. Cancer.

[11] Cottom H.E., Bshena F.I., Speight P.M., Craig G.T., Jones A.V. (2012). Histopathological features that predict the recurrence of odontogenic keratocysts. J. Oral Pathol. Med..

[12] Katabi N., Xu B. (2018). Salivary gland neoplasms: Diagnostic approach with focus on patterns of recognition and useful ancillary tools. Diagn. Histopathol..

[13] Satish V., Prabhadevi M.C., Sharma R. (2011). Odontome: A Brief Overview. Int. J. Clin. Pediatr. Dent..

[14] Jahagirdar P.B., Kale A.D., Hallikerimath S. (2015). Stromal characterization and comparison of odontogenic cysts and odontogenic tumours using picrosirius red stain and polarizing microscopy: A retrospective and histochemical study. Indian J. Cancer.

[15] Singh E., Pujari R.K., Murgod S., Girish H.C. (2016). Odontogenic tumour patterns: An introspective analysis. Br. J. Med. Res..

[16] Siwach P., Joy T., Tupkari J., Thakur A. (2017). Controversies in Odontogenic Tumours: Review. Sultan Qaboos Univ. Med. J..

[17] Su C.Y., Li J.Q., Zhang L.L., Wang H., Wang F.H., Tao Y.W., Wang Y.Q., Guo Q.R., Li J.J., Liu Y. (2020). The Biological Functions and Clinical Applications of Integrins in Cancers. Front. Pharmacol..

[18] Angadi P.V. (2011). Head and neck: Odontogenic tumour: Ameloblastoma Atlas Genet. Cytogenet. Oncol. Haematol..

[19] Sathi G.S., Fujii M., Tamamura R., Borkosky S.S., Katase N., Kawakami T., Nagatsuka H., Nagai N. (2008). Juxta-epithelial hyalinization inhibits tumour growth and invasion in ameloblastoma. J. Hard Tissue Biol..

[20] Dandriyal R., Gupta A., Pant S., Baweja H.H. (2011). Surgical management of ameloblastoma: Conservative or radical approach. Natl. J. Maxillofac. Surg..

[21] Li T., Wu Y., Yu S., Yu G. (2002). Clinicopathological features of unicystic ameloblastoma with special reference to its recurrence. Chin. J. Stomatol..

[22] Cadavid A.M., Araujo J.P., Coutinho-Camillo C.M., Bologna S., Junior C.A., Lourenço S.V. (2019). Ameloblastomas: Current aspects of the new WHO classification in an analysis of 136 cases. Surg. Exp. Pathol..

[23] Ahmed S.P., Gunasekaran N., Arunachalam P., Annasamy R.K. (2020). Mural unicystic ameloblastoma with multifarious histopathological patterns: An exquisite case report. Indian J. Dent. Res..

